# Validity and Reliability of Using a Self-Lavaging Device for Cytology and HPV Testing for Cervical Cancer Screening: Findings from a Pilot Study

**DOI:** 10.1371/journal.pone.0082115

**Published:** 2013-12-20

**Authors:** Heidi E. Jones, Mahesh M. Mansukhani, Guo-Xia Tong, Carolyn L. Westhoff

**Affiliations:** 1 Department of Obstetrics & Gynecology, Columbia University Medical Center, New York, New York, United States of America; 2 Epidemiology & Biostatistics Program, City University of New York School of Public Health and Hunter College, New York, New York, United States of America; 3 Department of Pathology and Cell Biology, Columbia University Medical Center, New York, New York, United States of America; Iran University of Medical Sciences, Iran (Islamic Republic of)

## Abstract

Self-sampling could increase cervical cancer screening uptake. While methods have been identified for human papillomavirus (HPV) testing, to date, self-sampling has not provided adequate specimens for cytology. We piloted the validity and reliability of using a self-lavaging device for cervical cytology and HPV testing. We enrolled 198 women in New York City in 2008–2009 from three ambulatory clinics where they received cervical cancer screening. All were asked to use the Delphi Screener™ to self-lavage 1–3 months after clinician-collected index cytological smear (100 normal; 98 abnormal). Women with abnormal cytology results from either specimen underwent colposcopy; 10 women with normal results from both specimens also underwent colposcopy. We calculated sensitivity of self-collected cytology to detect histologically confirmed high grade lesions (cervical intraepithelial neoplasia, CIN, 2+); specificity for histology-negative (CIN 1 or lower), paired cytology negative, or a third cytology negative; and kappa for paired results. One hundred and ninety-seven (99.5%) women self-collected a lavage. Seventy-five percent had moderate to excellent cellularity, two specimens were unsatisfactory for cytology. Seven of 167 (4%) women with definitive results had CIN2+; one had normal and six abnormal cytology results with the self-lavage (sensitivity = 86%, 95% Confidence Interval, CI: 42, 100). The kappa for paired cytology was low (0.36; 95% CI: 0.25, 0.47) primarily due to clinician specimens with atypical squamous cells of undetermined significance (ASC-US) and low grade squamous intraepithelial lesion (LSIL) coded as normal using Screener specimens. However, three cases of HSIL were coded as ASC-US and one as normal using Screener specimens. Seventy-three women had paired high-risk HPV tests with a kappa of 0.66 (95% CI: 0.49, 0.84). Based on these preliminary findings, a larger study to estimate the performance of the Screener for co-testing cytology and HPV or for HPV testing with cytology triage is warranted.

**Trial Registration:**

ClinicalTrials.gov NCT00702208

## Introduction

Approximately 50% of women enrolled in one of seven large private insurance plans and diagnosed with cervical cancer between 1995 and 2000 in the United States (US) had not been screened in the three years prior to diagnosis [1]. Under-screening is likely to be higher among uninsured women. Finding innovative ways to screen for cervical cancer could improve uptake among under-screened populations.

Offering self-sampling, in place of a pelvic examination, which some women find embarrassing or uncomfortable [2], [3], could increase screening uptake. A number of self-sampling methods such as tampon and swab provide valid specimens for human papillomavirus (HPV) tests [4]–[6]; however, to date, self-sampling has not been found to provide adequate specimens for cytology [7].

HPV tests will be increasingly used as the primary screening test [8], but cytology will continue to play a role. HPV tests are not useful for women under age 30 [8], given their high prevalence of transient infections [9]. Further, cytology can be used for triage of HPV-positive cases with its higher specificity until new diagnostics are fully developed [10]. Identifying a self-sampling method that can be used for both HPV testing and cytology is thus important for optimizing self-sampling options.

We piloted the validity and reliability of using a self-lavaging device, the Delphi Screener™ (Delphi Bioscience, Scherpenzeel, Netherlands), for cervical cytology by comparing paired self- and clinician-obtained specimens using liquid-based cytology (LBC) among 198 women, and high-risk HPV in a sub-sample.

## Materials and Methods

The protocol for this study and supporting Standards for reporting of Diagnostic Accuracy (STARD) checklist are available as supporting information (see Protocol S1 and Checklist S1).

### Recruitment and study visit

From December 1, 2008 to August 31, 2009, we enrolled 198 women who had attended one of three ambulatory clinics at the New York Presbyterian Hospital (NYPH) for cervical cancer screening. Clinicians at these three clinics asked women receiving cervical cancer screening as part of standard clinical care for permission to share their contact information with recruiters. Recruiters phoned a convenience sample of consenting women to schedule study visits or record reasons for ineligibility or declining participation. During the pre-study index visit, women underwent a standard pelvic examination with clinician-collected liquid based cytology (LBC), and HPV testing if ordered by the clinician.

Recruiters scheduled study enrollment visits 1–3 months after the index visit. One month after the index cytology results should have allowed sufficient time for cervical cells to replenish, with an upper limit of three months to minimize the potential for true changes in cervical cytology. By design, half of the participants had normal and half abnormal cytology results at the index visit to achieve the required sample size of 100 in each group. We used atypical squamous cells of undetermined significance (ASC-US) or worse to define abnormal cytology as these diagnoses trigger clinical follow-up [11]. Women whose index cervical screening results included co-testing with HPV were also co-tested with HPV using the Delphi Screener specimen. To be eligible, women needed to be at least 18 years old. Women were excluded if they reported current pregnancy, breastfeeding, hysterectomy, or discomfort reading on their own in Spanish or English. Women were asked not to schedule an appointment if they were within 4 days of the first day of their menses, or if they had undergone colposcopy prior to the study visit, as colposcopy and biopsy could change cytological results.

All participants provided written informed consent and received a packaged Screener for use in a private room at the clinical research site along with pictorial user instructions (which were also on the wall of the room in poster format). Study interviewers reviewed instructions with participants before they used the Screener on their own in a private room. The Delphi Screener is a sterile, plastic, syringe-like device containing 5 mL of buffered saline for self-lavage. The Screener currently does not have US Food and Drug Administration clearance. Columbia University's institutional review board approved use of the device as investigational with non-significant risk (ClinicalTrials.gov NCT00702208). Study implementation followed the ethical standards of the Declaration of Helsinki. Study visits during which women collected the self-lavage specimens took approximately 30–60 minutes and women received $30 to reimburse them for their time. Women were not reimbursed for follow-up visits that were part of their standard clinical care.

### Specimen handling

Staff delivered specimens to the laboratory within 24 hours. Laboratory staff centrifuged specimens at 1700 rpm for five minutes, pouring off the supernatant, and pipetting the cell pellet into a Cytyc PreservCyt ThinPrep vial with 20 mL of PreservCyt (Hologic, Marlborough, MA). Stability testing on nine pre-test specimens found no decrease in cellular integrity at 24 or 48 hours after receipt in the laboratory. Based on this stability testing, centrifugation and fixing was batched and completed within 72 hours of self-collection (up to 24 hours for delivery to the laboratory plus up to 48 hours from receipt to processing).

After suspension in PreservCyt, specimens were processed per manufacturing guidelines for ThinPrep specimens. Participants whose index screening included co-testing for HPV were co-tested using the Screener specimen with the Hybrid Capture II (Qiagen, Hilden, Germany) test per manufacturer specifications with a cut-off of one relative light unit.

Cytotechnologists used the 2001 Bethesda classification system [12] and recorded amount of fluid collected, cellularity, and presence of transformation zone cells (defined as endocervical and/or metaplastic cells). Each sample was reviewed by two cytotechnologists blinded to one another's diagnosis. These same cytotechnologists diagnosed all of the specimens collected using the self-lavage specimen. Abnormal slides were referred to a cytopathologist for final diagnosis. Cytotechnologists and cytopathologists were blinded to index results.

### Clinical follow-up

Participants with abnormal cytology result either from the pre-study index visit with standard clinician collection or using the self-lavaging device as part of the study were invited for colposcopy. Ascertainment bias occurs when only people with positive screening results are brought back for further diagnosis. Ten women (10%) with normal cytology results using both specimens were also invited for colposcopy to estimate ascertainment bias; these women received an additional $30 for this visit. Colposcopy was performed at three sites. All sites collected biopsies of acetowhite lesions. One site routinely collected endocervical curettings (ECCs), one collected ECC if no biopsy was taken, and one collected ECC if visualization of the transformation zone was incomplete, reflecting the lack of consensus on ECC utility when the transformation zone is fully visualized [13]. Women were followed through January 25, 2010.

### Sample size

The sample size was originally calculated using the index cytological smear as the gold standard; 98 women with normal/abnormal results would detect a sensitivity/specificity of 80% with a lower 95% confidence limit of 65% [14]. As cytology is known to have limited sensitivity [15], however, results from two or more biopsies should be used to define true precursors to cervical cancer [16]. Our pilot, proof-of-concept study was not powered based on biopsy results; seven participants had histologically confirmed high-grade lesions resulting in 29% power to detect a sensitivity of 80% with a lower 95% confidence limit of 65%.

### Statistical analyses

We calculated kappa statistics for clinician- and self-collected specimens for cytology and for HPV and estimated sensitivity and specificity, using colposcopy endpoints as the gold standard to define true cases. For this gold standard, true positives were defined as histologically confirmed high-grade cervical intraepithelial neoplasia (CIN2+) from biopsy or ECC. True negatives were defined as women who: 1. had normal cytology with both specimens but no colposcopy (n = 83); 2. had normal findings during colposcopy visit (defined as negative or low-grade biopsy/ECC or satisfactory colposcopy with no acetowhite lesions visualized, n = 70); or 3. missed colposcopy, but had normal repeat cytology after 6 months or more at the index clinic if ASC-US or low grade squamous intraepithelial lesions (LSIL) at index visit (n = 7). None of the ten women with normal results who underwent colposcopy to estimate ascertainment bias had abnormalities. Given that colposcopy was not used to confirm all negatives, we include adjusted estimates assuming that 1% of women [17] who had normal cytology with both specimens were missed cases of CIN2+. Additionally, we calculated secondary sensitivity and specificity estimates including CIN1 as true positives. We calculated 95% confidence intervals for sensitivity and specificity estimates using exact confidence intervals based on binomial probabilities.

We compared index cytology result by clinic, age and sexual activity. We compared age and initial cytology result of women who enrolled to those who refused or missed scheduled appointments and of the 167 women with validity results to those missing results. We used Fisher's exact tests to compare categorical outcomes and the Kruskal-Wallis test for non-parametric data to compare continuous outcomes. Analyses were conducted in Stata (StataCorp, College Station, TX).

## Results

During recruitment months, 5,509 women underwent cytological screening at participating clinics and 5,479 had sufficient samples. Of these women, 198 (3.6%) eligible women enrolled, 202 (3.7%) declined participation, 122 (2.2%) missed their study appointment, 18 were ineligible (0.3%) and most (4,939, 90.1%) were never invited to participate in this convenience sample (Figure S1). Thus 38% of those invited declined participation, primarily citing lack of time as their reason, and an additional 23% did not come to their study visit.

The majority of women were Latina (85%) and sexually active in the last month (76%) with a median age of 31 (Table 1). Nineteen percent of the participants were post-menopausal. Women with normal index cytology results using clinician-collected specimens were older than women with abnormal cytology (median age of 42 versus 24 years respectively, p<.001, Table 1). This pattern remained using the self-lavage cytology results; the median age for women with normal results on self-lavage was 32 versus 24 years for women with abnormal cytology, p = 0.01. Among the 98 women with abnormal cytology results, 54 (55%) had LSIL, 38 (39%) ASC-US, 4 (4%) high grade squamous intraepithelial lesion (HSIL), one (1%) atypical squamous cell cannot rule out HSIL (ASC-H) and one (1%) atypical glandular cells (AGC). The distribution of all abnormal results from participating clinics during recruitment months (n = 870) was similar: 46% LSIL, 48% ASC-US, 3% HSIL, 2% ASC-H and 1% AGC. Women who refused or missed their appointment were similar to participants for index cytology results, HPV results, and age. Women with missing versus definitive results (Figure S1) were also similar by age, index HPV, and index cytology. Results from colposcopy were used for definitive diagnosis for 46% (77/167) of women. The median time between index visit and colposcopy was 127 days (interquartile range, IQR: 69, 260).

**Table 1:** Demographic, sexual history and clinical characteristics by index cytology result; validity and reliability of the Delphi Screener for cervical cancer screening, New York City, 2009.

Characteristics	Index cytology result		Total (N = 198)	p-value*
	Normal (n = 100)	Abnormal (n = 98)		
**Self-reported race ethnicity, n(%)** **				0.06
Latina/Hispanic	81 (82.7)	85 (87.6)	166 (85.1)	
African American/Black	17 (17.3)	8 (8.3)	25 (12.8)	
Other	0 (0.0)	4 (4.1)	4 (2.0)	
**Median age in years**	42	24	31	0.00
(IQR/range)	(29–49/18–65)	(22–32/18–72)	(23–45/18–72)	
**Time of last sex, n(%)** ***				0.11
Within the last month	69 (69.7)	81 (82.6)	150 (76.1)	
Within the last year	14 (14.1)	8 (8.2)	22 (11.2)	
More than a year ago	16 (16.2)	9 (9.2)	25 (12.7)	
**Post-menopausal, n (%)** ***	30 (30.3)	8 (8.2)	38 (19.3)	0.00
**Clinician-collected cytological result, n (%)**				na
Normal	100 (100.0)	0 (0.0)	100 (50.0)	
ASC-US	0 (0.0)	38 (38.8)	38 (19.2)	
LSIL	0 (0.0)	54 (55.1)	54 (27.3)	
ASC-H	0 (0.0)	1 (1.0)	1 (0.5)	
AGC	0 (0.0)	1 (1.0)	1 (0.5)	
HSIL	0 (0.0)	4 (4.1)	4 (2.0)	
**Clinician-collected HPV result, n (%)**				0.00
Positive	4 (4.0)	26 (26.5)	30 (15.2)	
Negative	42 (42.0)	4 (4.1)	44 (23.2)	
Insufficient specimen	1 (1.0)	5 (5.1)	6 (3.0)	
Not done	53 (53.0)	63 (64.3)	116 (58.6)	
**Self-sampled cytological result, n (%)**				0.00
Normal	92 (92.0)	55 (56.1)	147 (74.2)	
ASC-US	4 (4.0)	14 (14.3)	18 (9.1)	
LSIL	1 (1.0)	26 (26.5)	27 (13.6)	
ASC-H	2 (2.0)	1 (1.0)	3 (1.5)	
AGC	0 (0.0)	0 (0.0)	0 (0.0)	
HSIL	0 (0.0)	0 (0.0)	0 (0.0)	
Insufficient specimen/not collected	1 (1.0)	2 (2.0)	3 (1.5)	
**Self-sampled HPV result, n (%)**				0.00
Positive	9 (9.0)	24 (24.5)	33 (16.7)	
Negative	34 (34.0)	7 (7.1)	41 (20.7)	
Insufficient specimen	2 (2.0)	0 (0.0)	2 (1.0)	
Not done	55 (55.0)	67 (68.4)	122 (61.6)	
**Colposcopy result, n (%)**				0.00
CIN 2+	0 (0.0)	7 (7.1)	7 (3.5)	
CIN 1	0 (0.0)	15 (15.3)	15 (7.6)	
Normal histology (biopsy/ECC)	5 (5.0)	35 (35.7)	40 (20.2)	
Normal colposcopy, no histology	5 (5.0)	10 (10.2)	15 (7.6)	
Not done (normal cytology/HPV)	83 (83.0)	7 (7.1)****	90 (45.5)	
Insufficient biopsy specimen	5 (5.0)	2 (2.0)	7 (3.5)	
Loss to follow-up	1 (1.0)	20 (20.4)	21 (10.6)	
No/unsatisfactory lavage result	1 (1.0)	2 (2.0)	3 (1.5)	

na = not applicable; IQR = interquartile range; HPV = human papillomavirus; ASC-US = atypical squamous cells of undetermined significance; ASC-H = atypical squamous cells cannot rule out high grade; LSIL = low grade squamous intraepithelial lesion; AGC = atypical glandular cells; HSIL = high grade squamous intraepithelial lesion; CIN = cervical intraepithelial lesion; ECC = endocervical curetting.

* p-values are calculated using the Fisher's exact tests to compare proportions and the Kruskal Wallis test to compare medians.

** N = 195, missing data on two women with normal and one with abnormal index cytology.

*** N = 197, one woman with normal index cytology missing all demographic data other than age.

**** Received a second clinician-collected cytology test per standard clinical care which was normal.

### Specimen collection and quality

Only one morbidly obese participant was unable to self-lavage, resulting in 197 specimens. The median fluid collected was 1.0 mL, ranging from 0.1 to 5.0 mL. Cytotechnologists coded 75% of specimens as having moderate to excellent cellularity, 23% low cellularity and 2% scant. Two of the Screener specimens (1%) were unsatisfactory for cytology, compared to 0.5% (30/5509) of clinician-collected specimens from participating clinics during the same time period (z-test, p = 0.51). A total of 195 self-lavage specimens were collected and readable for cytology.

The median number of days between clinician- and self-collected specimens was 60 days; the median for women whose index cytology was normal was longer than women with abnormal results (65 versus 55 days respectively, p = 0.02). Transformation zone cells were present on 93% of clinician-collected specimens compared to 18% of Screener specimens (p<.001). Transformation zone cells were less likely to be present using clinician-collected specimens for post-menopausal women: 81% of 38 post-menopausal women had transformation zone cells compared to 96% of 159 menopausal women (p = 0.006). However, the reverse was true using self-lavage specimens, 26% of the post-menopausal specimens showed transformation zone cells compared to 16% of the menopausal women (p = 0.01).

### Delphi Screener specimens for cytology and HPV

Most women (92/99, 93%) whose index cytology was normal were diagnosed as normal using the Screener (Table 2). Paired specimens for dichotomous cytology results showed 68% overall agreement, with a kappa of 0.36 (95% Confidence Interval, CI: 0.25, 0.47). This result did not change when limited to women with 28–60 days between specimen collections (n = 93, kappa = 0.38, 95% CI: 0.21, 0.55). However, agreement was greater for women over the age of 30 (n = 99, kappa = 0.48, 95% CI: 0.29, 0.67) than for women aged 30 and under (n = 97, kappa = 0.20, 95% CI: 0.07, 0.34).

**Table 2:** Number of women with results from index clinician-collected specimens by results from Screener specimen and kappa statistic; validity and reliability of the Delphi Screener for cervical cancer screening, New York City, 2009.

	Cytology result using clincian-collected specimen (n = 195*)					
Cytology result using Screener specimen	Normal	ASC-US	LSIL	ASC-H	AGC	HSIL
**Normal**	**92**	28	25	0	1	1
**ASC-US**	4	**3**	8	0	0	3
**LSIL**	1	6	**20**	0	0	0
**ASC-H**	2	0	0	**1**	0	0
**AGC**	0	0	0	0	**0**	0
**HSIL**	0	0	0	0	0	**0**
**Weighted kappa** **	0.34					
**(95% CI)**	(0.24, 0.45)					

	HPV result using Digene Hybrid Capture II (n = 73***)	
HPV result using Screener specimen	HPV negative	HPV positive
**HPV negative**	**36**	5
**HPV positive**	7	**25**
**Kappa**	0.66	
**(95% CI)**	(0.49, 0.84)	

CI = confidence interval; HPV = human papillomavirus; ASC-US = atypical squamous cells of undetermined significance; ASC-H = atypical squamous cells cannot rule out high grade; LSIL = low grade squamous intraepithelial lesion; AGC = atypical glandular cells; HSIL = high grade squamous intraepithelial lesion.

* Excluding 2 unsatisfactory specimens, 1 originally ASC-US/1 originally LSIL.

** Weighted Kappa was calculated by combining ASC-H, AGC, and HSIL into uppermost category, and assuming 1 point difference between each category.

*** 1 specimen was unsatisfactory using clinician specimen, 2 specimens were unsatisfactory using Screener specimen.

As seen in Figure S1, 167 women were included in the estimates of sensitivity and specificity; 28 women with valid Screener cytology results did not come for their scheduled colposcopy visit or had unsatisfactory colposcopy results. Seven women had histologically confirmed high-grade lesions; all had abnormal clinician-collected cytology: one HSIL, four LSIL, one ASC-H and one ASC-US. Five of seven had identical self-collected cytology results. One woman diagnosed as HSIL using the clinician-collected specimen was diagnosed with ASC-US using the Screener specimen and one diagnosed as ASC-US was diagnosed as normal with the Screener.

Using unadjusted data, clinician-collected cytology had a 7/7 or 100% sensitivity (97.5% CI: 59, 100) and a specificity of 93/160 or 58% (95% CIs: 50, 66), while the Screener had a sensitivity of 6/7, 86% (95% CIs: 42, 100) and a specificity of 128/160, 80% (95% CIs: 73, 96) to identify CIN 2+ (Table 3). Adjusting for ascertainment bias, the sensitivities are 87% (95% CIs: 47, 100) for clinician-collected cytology and 75% (95% CIs: 35, 97) for self-lavage cytology; the specificities are 58% (95% CIs: 50, 66) and 80% (95% CIs: 73, 86) respectively.

**Table 3:** Sensitivity and specificity of cytology for clinician-collected and Screener specimens; validity and reliability of the Delphi Screener for cervical cancer screening, New York City, 2009.

		Colposcopy/repeat cytology (n = 167)	
Unadjusted		High grade (CIN 2+)	Normal
Clinician-collected cytology	Abnormal	7	67
	Normal	0	93
	Total	7	160
	Sensitivity/Specificity	100.0	58.1
	(95% CI)*	(59.0, 100)**	(50.0, 65.9)
Screener-collected cytology	Abnormal	6	32
	Normal	1	128
	Total	7	160
	Sensitivity/Specificity	85.7	80.0
	(95% CI)*	(42.1, 99.6)	(72.9, 85.9)
**Adjusted for ascertainment bias** ***			
Clinician-collected cytology	Abnormal	7	67
	Normal	1	92
	Total	8	159
	Sensitivity/Specificity	87.5	57.9
	(95% CI)*	(47.3, 99.7)	(49.8, 65.7)
Screener-collected cytology	Abnormal	6	32
	Normal	2	127
	Total	8	159
	Sensitivity/Specificity	75.0	79.9
	(95% CI)*	(34.9, 96.8)	(72.8, 85.8)

CI = confidence interval.

* 95% Confidence intervals are calculated using exact confidence intervals based on binomial probabilities.

** One-sided 97.5% confidence interval based on binomial probabilities.

*** The adjusted assumes that one case of a high-grade lesion was misclassified as normal by both types of specimens.

Including histologically confirmed CIN1 as true positives does not change these results substantially. Fifteen cases of low-grade histology were identified; all fifteen had abnormal cytology using the clinician-collected specimen (twelve LSIL, two ASC-US, one HSIL) and eleven had abnormal cytology using the Screener (six LSIL, five ASC-US). For the clinician-collected specimens, this definition results in a sensitivity of 22/22, or 100% (97.5% CI: 85, 100) and specificity of 52/145, or 64% (95% CI: 56, 72). For Screener specimens, using this definition, sensitivity is 17/22, 77% (95% CI: 55, 92) and specificity is 124/145, 86% (95% CI: 79, 91).

Seventy-six Screener specimens (39%) were co-tested for HPV. Two (3%) were insufficient for HPV diagnosis, compared to 9% (174/1918) for clinician-collected specimens during the same time period (z-test, p = 0.03). Overall HPV agreement between clinician- and self-collected specimens was 84% with a kappa of 0.66 (95% CIs: 0.49, 0.84, Table 2).

## Discussion

Delphi Screener specimens showed moderate to high cellularity and comparable rates of sufficient specimens to clinician-collected specimens for cytology and HPV. The main difference in specimen quality was the presence of transformation zone cells: over 90% of clinician-collected specimens contained these cells compared to 18% of Screener specimens. Cross-sectional studies have found an association between presence of these cells, especially endocervical cells, and concurrent abnormal cytological findings [18]. Longitudinal studies, however, have found no association between the absence of transformation zone cells among women with negative cytology and subsequent high grade lesions [19], [20]. Furthermore, the validity of HPV testing is independent of the presence of transformation zone cells [21]. Therefore, the absence of these cells may not be an important indicator of specimen quality for use in cervical cancer screening. However, the utility of transformation zone cells for women 30 years of age and under has not been well-studied.

Agreement between the clinician- and Screener-collected cytology result was low (kappa = 0.36), primarily due to women with ASC-US and LSIL from clinician-collected specimens diagnosed as normal using Screener specimens. Agreement for cervical cytology with clinician-collected specimens also tend to be low; one study reported kappas of 0.26 to 0.40 among six cytologists reading the same 70 slides for specimen adequacy alone [22]. Another study of 117 abnormal slides, found kappas of 0.39 to 0.57 among seven cytologists, depending on classification system used [23]. The fairly low kappa in this study may be an indicator of low reliability for cervical cytology in general rather than low reliability from using the Screener specimen, although a larger study is needed to ensure that high grade cases are not systematically missed.

As expected women who had abnormal cytology results (primarily ASC-US and LSIL) were, on average, younger than women with normal cytology results. The kappa for cytology improved with older age, as would be anticipated given fewer abnormal findings. Future studies should be designed to produce separate estimates of the performance of self-lavage for women aged 30 years and under versus over 30.

Agreement of high-risk HPV testing between the Screener and clinician-collected specimen was moderate (kappa = 0.66), and comparable to an earlier study using the GP5+/6+ polymerase chain reaction HPV test with the Screener (kappa = 0.71) [24]. These findings are also similar to estimates for self-collected tampons or swabs for HPV testing in a recent meta-analysis (kappa = 0.66) [6], suggesting the Screener is comparable to other self-sampling methods for high-risk HPV testing.

Clinically, more important than agreement between the two specimens is their ability to detect abnormalities in women with CIN2+. Our preliminary point estimate of the sensitivity of the Screener using cytology was good even after adjusting for possible ascertainment bias (75.0%), although the 95% confidence interval was wide with a lower bound of 35%. The point estimate for the specificity of the Screener specimen for cytology was higher than that of clinician-collected specimens. While these preliminary estimates are promising, a larger study is needed to better estimate the extent to which high grade cases might be missed before relying on a Screener specimen alone for cytology.

The idea to use self-lavage for cytology is not new. In the 1960s, a number of studies tested the use of the Davis cytopipette or irrigation smear for self-lavage for cytological smears [25]–[29]. While initial results were promising [26], ultimately high proportions of unsatisfactory specimens, as high as 37% [29], coupled with low sensitivity, prohibited further use of the device in cervical cancer screening. In one study, 5 of 13 histologically confirmed cervical cancer cases tested negative using the irrigation smear [29]. In the current study, however, six of the 7 histologically confirmed cases of CIN2+ and 11 of the 15 histologically confirmed cases of CIN1 had abnormal cytology results using the self-lavage, suggesting further study of the Screener for cytology is warranted. Further, most women (79%) reported preferring self-lavage over clinician-collection for future cervical cancer screening [30].

The present study had a number of limitations. The study was not sufficiently powered based on colposcopy results for endpoints; this study tested proof-of-concept for use of Screener self-lavage specimens for cervical cytology. Colposcopy was not used on all women, and sixteen women who received colposcopy did not have a specimen taken for histology. Additionally women were not followed for a sufficiently long time to produce robust specificity estimates; clearly some high grade lesions may have been missed. Missing more than 1% of negatives is highly unlikely in this low-risk population however, and adjusted sensitivity and specificity calculations for the Screener specimen remain reasonable. Finally, while the same two cytotechnologists diagnosed all of the self-lavage specimens, index clinician-collected specimens were diagnosed by a larger pool of cytotechnologists available at the hospital laboratory; some disagreements in cytological findings could be caused by this difference in cytotechnologists.

Despite the low reliability of cytology between clinician- and self-collected specimens, most high-grade lesions were caught and reliability of HPV testing was good. A larger study to estimate the performance of the Screener for HPV testing with cytology triage or co-testing is warranted and could result in identifying an important new tool to increase cervical cancer screening uptake in hard-to-reach populations. Cost of the device would also need to be considered prior to large-scale introduction.

## Supporting Information

Figure S1 **Participant flow; validity and reliability of the Delphi Screener for cervical cancer screening, New York City, 2009.**
(TIFF)

Protocol S1 **Protocol for, “The feasibility and acceptability if using the Pantarhei Screener for cervical cytology testing among low income women in New York City,” reviewed and approved by the Columbia University Medical Center's Institutional Review Board.**
(PDF)

Checklist S1 **STARD Checklist for reporting of studies of diagnostic accuracy.**
(PDF)
