# Supplementary material for: Validity and Reliability of Using a Self-Lavaging Device for Cytology and HPV Testing for Cervical Cancer Screening: Findings from a Pilot Study
Source: PLoS One. 2013 Dec 20;8(12):e82115. doi: 10.1371/journal.pone.0082115 (PMC3869665; doi:10.1371/journal.pone.0082115)
Supplement: Protocol S1 — Protocol for, “The feasibility and acceptability if using the Pantarhei Screener for cervical cytology testing among low income women in New York City,” reviewed and approved by the Columbia University Medical Center's Institutional Review Board. (PDF) [file pone.0082115.s002.pdf]

Columbia University Human Subjects Protocol Data Sheet

Protocol: IRB-AAAD1382(Y2M01) Modification **Protocol Status: Approved** **Effective Date: 07/09/2009**
Expiration Date: 04/09/2010

Originating Department: **OBSTETRICS AND GYNECOLOGY (538)**
Submitting To: **Medical Center**
Title: **The feasibility and acceptability of using the Pantarhei Screener for cervical cytology testing among low income women in New York City**
Sponsor Protocol Version#:
Abbreviated title: **Feasibility and acceptability of the Screener for cytology**
IRB of record: **Columbia University Medical Center**
IRB number used by the IRB of record:

Affiliated Institutions: -Standard Columbia Submission
Protocol Begin Date: 05/01/2008
Protocol End Date: 09/30/2009

Personnel Staff

Name/Phone	Role	COI-Date	Courses	Dept	Edit/ View	Procedure Experience
*Carolyn Westhoff, (clw3) 212-305-4805 Prof At Cpmc	Principal Investigator	02/06/2009	1. HS GCP - Patient/Clinical - 12/22/1999 2. HIPAA: Health Insurance Portability Accountability Act Research Training Course - 01/19/2004 3. Research with Minors - 11/30/2007	538	Edit	
Gabriella Barbiero, (gb2189) 212-305-2360 Supervisor of Cytology	Technician	02/26/2009	1. HS GCP - Patient/Clinical - 02/26/2009 2. HIPAA: Health Insurance Portability Accountability Act Research Training Course - 02/26/2009	548	View	
Karen Brudney, (kfb2) 212-305-8507 Asst Clin Prof	Co-Investigator	08/28/2008	1. HS GCP - Patient/Clinical - 12/13/2000 2. HIPAA: Health Insurance Portability Accountability Act Research Training Course - 12/30/2003	527	View	
Paula Castano, (pc2137) 212-305-4805 Assistant Clinical	Study Physician	04/02/2009	1. HS GCP - Patient/Clinical - 08/05/2003 2. HIPAA: Health Insurance Portability Accountability	538	View	

Columbia University IRB
Approved for use until: 04/09/2010

Professor			Act Research Training Course - 05/18/2004<br>3. Research with Minors - 05/01/2007		
Sarah Goldsberry, (sg2616) 212-305-6098	Research Assistant	08/27/2008	1. HS GCP - Patient/Clinical - 08/22/2008<br>2. HIPAA: Health Insurance Portability Accountability Act Research Training Course - 08/22/2008<br>3. Research with Minors - 03/24/2009	538	View
Heidi Jones, (hej2103) 212-305-3732	Co-Investigator	02/17/2009	1. HIPAA: Health Insurance Portability Accountability Act Research Training Course - 11/01/2006<br>2. HS GCP - Patient/Clinical - 01/24/2007<br>3. HS GCP - Epid. & Soc. Behav. - 01/25/2007<br>4. Research with Minors - 02/12/2007	538	Edit
	Initiator				Edit
Annie Kao, (ack2127) 4086600378	Research Assistant	02/27/2009	1. HIPAA: Health Insurance Portability Accountability Act Research Training Course - 01/19/2009<br>2. HS GCP - Patient/Clinical - 01/22/2009<br>3. HS GCP - Epid. & Soc. Behav. - 01/22/2009	478	View
Rafael Lantigua, (ral4) 212-3056262 Professor Clin	Co-Investigator	01/23/2009	1. HS GCP - Patient/Clinical - 12/13/2000<br>2. MS Human Subjects - 04/04/2002<br>3. HIPAA: Health Insurance Portability Accountability Act Research Training Course - 09/09/2004	527	View

Columbia University IRB
Approved for use until: 04/09/2010

Mahesh Mansukhani, (mm322) 212-305-2646 Asst Prof	Co-Investigator	04/12/2009	1. HS GCP - Patient/Clinical - 01/31/2001<br>2. HIPAA: Health Insurance Portability Accountability Act Research Training Course - 01/14/2005<br>3. Research with Minors - 01/03/2008	548	View
Elizabeth Mayeda, (erm2127) 831-345-5215	Research Assistant	03/26/2009	1. HIPAA: Health Insurance Portability Accountability Act Research Training Course - 03/27/2008<br>2. HS GCP - Epid. & Soc. Behav. - 03/27/2008<br>3. Research with Minors - 03/26/2009	538	View
Emilee Pressman, (ejp2115) 2123054938	Research Assistant	03/10/2009	1. HIPAA: Health Insurance Portability Accountability Act Research Training Course - 03/18/2008<br>2. HS GCP - Patient/Clinical - 03/19/2008<br>3. HS GCP - Epid. & Soc. Behav. - 03/20/2008	538	View
Irene Raju, (ir77) 646-423-0244 Research Coordinator	Study Coordinator	11/07/2008	1. HIPAA: Health Insurance Portability Accountability Act Research Training Course - 09/30/2007<br>2. HS GCP - Patient/Clinical - 09/30/2007	538	Edit
Mariaelena Ramos, (mr2763) 212-305-3732	Recruitment Coordinator	07/22/2008	1. HS GCP - Patient/Clinical - 07/25/2007<br>2. HIPAA: Health Insurance Portability Accountability Act Research Training Course - 07/25/2007<br>3. Research with Minors - 01/23/2008	538	View
Maria Rodriguez, (mcr2140)	Research Assistant	04/07/2009	1. HS GCP - Patient/Clinical - 12/13/2007	538	View

Columbia University IRB
Approved for use until: 04/09/2010

212-444-8509			2. HIPAA: Health Insurance Portability Accountability Act Research Training Course - 12/14/2007<br>3. Research with Minors - 03/25/2009		
Linette Rosario Tejeda, (lr2488) 6468947297	Research Assistant	02/26/2009	1. HIPAA: Health Insurance Portability Accountability Act Research Training Course - 02/26/2009<br>2. HS GCP - Patient/Clinical - 02/26/2009	538	View
Guo-Xia Tong, (gt2125) 212-342-3910	Co-Investigator	05/28/2009	1. HIPAA: Health Insurance Portability Accountability Act Research Training Course - 09/02/2008<br>2. HS GCP - Patient/Clinical - 09/02/2008	548	View
Anupama Torgal, (at2412) 212-305-0947 Research Coordinator	Research Assistant	11/10/2008	1. HIPAA: Health Insurance Portability Accountability Act Research Training Course - 12/27/2006<br>2. HS GCP - Patient/Clinical - 01/05/2007<br>3. Research with Minors - 05/07/2009	538	View
Mary Wheat, (mw219) 212-3423941 Assistant Professor of Clinical Medicine	Co-Investigator	02/04/2009	1. HIPAA: Health Insurance Portability Accountability Act Research Training Course - 08/26/2004<br>2. HS GCP - Patient/Clinical - 09/09/2004	527	View
Teresa Wood, (tmw17) 342-1939	Co-Investigator	05/27/2009	1. HIPAA: Health Insurance Portability Accountability Act Research Training Course - 11/12/2008<br>2. HS GCP - Patient/Clinical - 11/13/2008	548	View

Columbia University IRB
Approved for use until: 04/09/2010

Modification

Current Number of Participants in Study:	0
Subject Enrollment Status:	Recruitment/Related Interventions Ongoing
Summary of Proposed Changes:	In response to the IRB's correspondence on this modification, we have made the following changes to the Informed Consent Form. These changes have been made to both the English and Spanish versions of the consent forms. Spanish translations were made as described in the memo originally attached to this modification of the protocol:

1. We edited the text in the "What is Involved in the Study?" section (or Study Procedures section) by:

- Clarifying in the first paragraph that a colposcope is used to look inside the vagina and any biopsies taken would be taken from the cervix.
- Adding to the third paragraph the following sentence, "If any of your test results are abnormal the most common next step would be to undergo colposcopy," to clarify that this procedure is not experimental but rather the standard of care.
- Adding to the end of the fourth paragraph the following text, "If you are invited for a second visit, this visit would involve a pelvic exam as you had when you got your original Pap, but will include using a colposcope, which is a magnifying lens with a light that the doctor will use to look inside of your vagina. If the doctor sees anything abnormal, he or she will discuss with you the possibility of taking a biopsy of the cervix."

2. Added the following paragraph to the "What are the Risks of the Study?" section:

If you come for a second visit to receive colposcopy, this 15-20 minute procedure involves discomfort similar to a pelvic examination for a Pap smear. During the procedure, the doctor may recommend taking a small biopsy of the cervix. Even with the use of local anesthesia, the biopsy may involve additional discomfort and may result in bleeding or spotting for a few days after the exam.

Below is the original text of the correspondence for this modification:

We propose these changes to the protocol:

1. Per the IRB's request, we have added the Cancer Center as a research site.

2. We have changed the protocol end date from June 2009 to May 2010 to allow time to complete enrollment (anticipated through August 2009) and data analysis (anticipated through May 2010).

3. We have changed the eligibility criteria to include that a woman cannot have colposcopy prior to enrollment. Colposcopy can change Pap results, and therefore would make a comparison of Pap results before and after colposcopy not valid. This has been updated under "7.

Columbia University IRB
Approved for use until: 04/09/2010

Study Participants" in the Study Description section in RASCAL. We have also added this to question 10 of the revised Screening Form (attached).

4. Enrollment has been slower than anticipated. We are therefore adding two new recruitment sites: 1. Student Health Services (SHS) and 2. the Family Planning & Preventive Services doctors' private office in the Herbert Irving Pavilion.

We have made the following changes to reflect the addition of these sites:

- added both locations to RASCAL research sites

- updated the first sentence of the third paragraph of the scientific and lay abstracts in RASCAL

- added Mary (Polly) Wheat, Medical Director of SHS, to the personnel as a co-investigator in RASCAL

- updated the first sentence of the paragraph under "Study Design and Statistical Procedures" in the Study Description section on RASCAL

- attached letters of support from Polly Wheat, Medical Director of SHS, and Carolyn Westhoff, Medical Direct of the FPPS private office

- attached a recruitment letter for SHS participants. Students attending the SHS for a normally scheduled Pap visit will be given the recruitment letter by their doctor. If they are interested in being contacted about the study, they will sign the recruitment letter and provide a phone number. Study staff will only call students who have signed the letter stating they are interested in participating.

- updated the ethnicity section of our study population in RASCAL by increasing estimated percent Caucasian from 0 to 10% and decreasing the estimated percent Hispanic from 85 to 75%

- updated the special populations in RASCAL to include Columbia University students

- updated the Subject Population Justification to describe the two new sites

- updated the Informed Consent Form section entitled "Why is this study being done?" to include the new sites.

5. We simplified the protocol. All study visits occur in the Presbyterian Hospital on the 16th floor in dedicated research space as opposed to at participating clinics. This has been updated in the Study Description section of RASCAL under "3. Study Procedures" as follows: "Interested women will come to the clinical research facility at the Division of Family Planning & Preventive Services in the Presbyterian Hospital's Department of Obstetrics & Gynecology, for

Columbia University IRB
Approved for use until: 04/09/2010

their single study visit, pending any abnormalities."

6. We propose offering colposcopy to more women in our study. Colposcopy is the gold standard for cervical cancer screening. We need colposcopy results to assess the validity and reliability of the study Pap results. The standard of care for women with abnormal Pap results is to either offer them a repeat Pap in 6-12 months or immediate colposcopy. The Family Planning Clinic, given current allocation of resources, refers many women with abnormal results to a repeat Pap in 6-12 months rather than immediate colposcopy. We will offer these women immediate colposcopy at the dedicated research site as part of the study. This will allow these women to know their health status sooner. They will not receive additional reimbursement for this study visit. If the woman has health insurance, her insurance will cover the cost of colposcopy as recommended standard of care. If the woman is uninsured or her insurance will not pay for colposcopy, the study will cover the cost of this procedure. All of these women will be given a modified informed consent form to sign (attached).

We also propose inviting 10 women with normal Pap results for colposcopy to assess ascertainment bias (false negative results). The Pap test is known to have a low sensitivity and therefore misses many cases. If the clinician sees anything abnormal using the colposcope, s/he will discuss the possibility of taking a biopsy with the woman. If nothing abnormal is seen, no biopsies will be taken. These women will receive an additional $30 for this visit. The study will cover the cost of any biopsies taken. We will recruit these women from newly enrolled women who have signed the new informed consent form.

This change has been updated as follows:

- added a sentence to Subject Compensation in RASCAL to read, "They receive an additional $30 if they are among the 10 women with normal pap smears and Screener test results who agree to a separate colposcopy study visit."

- Under "3. Study Procedures" in the Study Description section of RASCAL, we have clarified that it is a single study visit, pending any abnormalities and have updated the following sentence, "Finally, the research assistant will assist women with abnormal results in making appointments with the colposcopy clinic, further encourage them to maintain existing appointments, or arrange for them to return to the research site to receive colposcopy."

- On the Informed Consent Form in the Section entitled "Additional Costs," we have added the following sentence, "If your original pap was abnormal and you are unable to get immediate colposcopy through your clinic, we may also offer you a colposcopy exam at the research site. If you have health insurance, your insurance will pay for this visit. If you do not have health insurance, we will pay for the cost of this visit; you will not be responsible for any of the cost related to this visit."

Columbia University IRB
Approved for use until: 04/09/2010

- On the Informed Consent Form in the Section entitled "What is involved in this study?" We have added the following sentence to the first paragraph, "We will invite 10 women with normal results for a second visit which includes a pelvic exam and colposcopy; participating in this second visit is voluntary. This second visit would involve a pelvic exam as you had when you got your original Pap, but will include using a colposcope, which is a magnifying lens with a light that the doctor will use to look inside your vagina. If the doctor sees anything abnormal, he or she will discuss the possibility of taking a biopsy with you. It is your decision whether you wish to have a biopsy taken. We will pay for the cost of this visit and any biopsies taken; you will not be responsible for any of the cost related to this visit." We have also added the following sentence to the last paragraph in this section, "Taking part in this study will last 30-60 minutes and will generally include only this one study visit. If however, you have abnormal pap results you may be invited for a second visit, and 10 women with normal pap results will be invited for a second visit."

- On the Informed Consent Form in the section entitled, Compensation, we have added the following sentence, "If you are one of the 10 women with a normal pap result who agrees to come for a second visit for colposcopy, you will receive an additional $30."

7. All changes made to the English Informed Consent Form have also been made to the Spanish version of the Informed Consent Form (see attached memo describing the translation process and revised Spanish consent form).

Research

Research procedures:	Collection of Bodily Materials (other than blood)<br>Interview/Survey/Questionnaire<br>Device Study
Research facilities:	Cancer Center<br>New York Presbyterian Hospital - Columbia

<u>Research question(s):</u>

1. Primary Objective

   To investigate the feasibility of using the self-administered Pantarhei Screener for cervical cytology compared to the gold standard, clinician-collected spatula and brush with Thin Prep, by calculating the kappa, percent agreement, sensitivity, specificity, positive and negative predictive values
2. Secondary Objectives

   To evaluate the acceptability of the Pantarhei Screener compared to the pelvic examination with a speculum, plastic spatula and cervical brush.
3. To test the clarity of user instructions for the Pantarhei Screener.
4. To measure the variation in the amount of fluid collected using the Pantarhei Screener and to quantify the amount of cervicovaginal cells collected.
5. To test the feasibility of using Screener-obtained specimens for both cytology and HPV among women whose initial cytology included an HPV test and to compare HPV results to the gold standard clinician-collected specimens by calculating the kappa, percent agreement, sensitivity, and specificity.

Columbia University IRB
Approved for use until: 04/09/2010

6. To determine the percentage of indeterminate or insufficient specimen collections for the Screener compared to clinician-collected specimens in combination with cytology.
7. To compare the sensitivity and specificity of the Pantarhei Sampler versus clinician-collected specimens for detecting high grade squamous intraepithelial lesions (HSIL), as confirmed by colposcopy and biopsy among women with an abnormal cytology from the Screener-obtained and/or clinician-collected specimen
8. To record any adverse event that occurs from use of the device, as a measurement of safety.

Scientific abstract: The aim of this study is to evaluate the feasibility of using a novel self-lavaging device, the Pantarhei Screener, in combination with cytology for the detection of cervical abnormalities, compared to the gold standard, clinician-collected endocervical specimens, in terms of kappa, percent agreement, sensitivity, specificity, positive and negative predictive values. Women will answer questions about acceptability of the device and clarity of user instructions.

The Pantarhei Screener is a sterile, plastic, syringe-like device containing buffered saline which allows a woman to collect her own vaginal lavage. The device has approval for commercial use in Europe and much of Asia, with a Class 1 CE mark. The device was designed by gynecologists to fit a woman's body. The atraumatic shape precludes the potential for injuries from using the device, making it a device with non-significant risk.

210 women who have had a cervical cytology test at the Family Planning Clinic (including private offices), Infectious Diseases Clinic, Associates for Internal Medicine or Student Health Services in the last 1-3 months will be enrolled. All women will self-collect a specimen using the Pantarhei Screener and answer a set of questions on the acceptability of the self-sampler and the pelvic exam, clarity of user instructions, as well as baseline socio-demographic characteristics. Clinical care of women will be based on their standard cytology results, unless results from the Screener are abnormal, and those of the clinician-obtained specimen are normal; these women will be followed to ensure abnormalities were not missed on the standard test.

Lay abstract: The aim of this study is to see whether a new self-sampling device, the Pantarhei Screener, can be used for cervical cancer screening. The results using the new device will be compared to results using the current gold standard. The gold standard is clinician-collected endocervical specimens in combination with Thin Prep (often referred to as a 'Pap smear'). Additionally, women will be asked about the acceptability of using the device and how easy it is to understand the user instructions.

The Pantarhei Screener is a sterile, plastic, syringe-like device containing buffered saline which allows a woman to collect her own vaginal lavage (to 'self-squirt'). The device has approval for commercial use in Europe and much of Asia, with a Class 1 CE mark. The device was designed by gynecologists to fit a woman's body. The rounded shape precludes the potential for injuries from using the device, making it a device with non-significant risk.

210 women who have had a cervical cytology test at the Family Planning Clinic (including private offices), Infectious Diseases Clinic, Associates for Internal Medicine or Student Health Services in the last 1-3 months will be enrolled. All women will self-collect a specimen using the Pantarhei Screener and will be asked a set of questions on the acceptability of the self-sampler and the pelvic exam, clarity of user instructions as well as baseline socio-demographic characteristics. Clinical care of women will be based on their standard Pap results, unless results from the Screener are abnormal, and those of the Pap are normal; these women will be followed to ensure abnormalities were not missed on the Pap.

Funding

Funding Type	Source	Source Identifier	Rascal Proposal
External Medical Device	Pantarhei Devices		PT-AAAF1621

Location

Columbia University IRB
Approved for use until: 04/09/2010

Site	Building	Floor	Room
Associates for Internal Medicine (AIM) Clinic	Vanderbilt Clinic	2nd floor	
Infectious Diseases Clinic	Harkness Pavillion	6	
Family Planning Clinic	21 Audubon	1st	
Family Planning & Preventive Services Clinical Research Site	Presbyterian Hospital	16	16-80
Student Health Services	Bard Hall	1st floor	
Family Planning & Preventive Services Private Office	Herbert Irving Pavilion	4th floor	

Subjects

Total Number of Subjects:	210
Columbia University/NY Presbyterian Hospital Outpatient Subjects:	210
Columbia University/NY Presbyterian Hospital Inpatient Subjects:	0

Population Gender		Population Age		Population Ethnicity	
Females	100%	18-65	100%	African-American	10%
				Asian	3%
				Caucasian	10%
				Hispanic	75%
				Other	2%

Special Populations: Non-english speaking
CU Students
Economically disadvantaged

Recruitment Media: Person to Person
Telephone
Flyer/Handout
Direct Mail

Subject justification: Each of the five participating clinics represents unique client populations; young women attend the Family Planning Clinic, Student Health Services and FPPS private offices, middle age and older women attend the Associates for Internal Medicine, and women attending the Infectious Disease Clinics who require more frequent monitoring of cervical cytology. Including the five clinics in this study will allow us to obtain a varied view of the feasibility and acceptability of using the device. Additionally, all five clinics would benefit in terms of clinic efficiency and reduced loss to follow up of patients if the device were to prove feasible and acceptable to use in replace of the pelvic exam.

Subject compensation: Participants receive $30 for participating in the study. They receive an additional $30 if they are among the 10 women with normal pap smears who agree to a separate colposcopy study visit.

Columbia University IRB
Approved for use until: 04/09/2010

Compensation justification: Participants come to the study venue solely for the purpose of participating in the research study; they will not receive direct medical benefit beyond additional counseling on cytology results and facilitation of colposcopy appointments if indicated. As such, they will need to be compensated for the time and effort involved in coming to the study venue. The procedures upon arrival should not take longer than half an hour.

Consent form waiver/alteration request:

Recruitment url:

Investigational Product

Type	Name	Description
Device	Pantarhei Screener	The Pantarhei Screener is a sterile, plastic, syringe-like device containing 5 mls of buffered saline that allows a woman to collect her own vaginal lavage. The device looks like a plastic tampon applicator. The device was designed by gynecologists to fit a woman's body. The atraumatic shape precludes the potential for injuries from using the device, making it a device with non-significant risk. The Screener currently has a CE label for Class 1 sterilized products allowing it to be used on a commercial basis in Europe and many countries in Asia.<br><br>Data to date suggest that the device provides a high quality specimen for the detection of high-risk HPV. In a study among 96 women in the Netherlands, the Screener had 87% agreement with a kappa of 0.71 compared with an endocervical brush obtained by a clinician in combination with the GP5+/6+ polymerase chain reaction (PCR) test to detect high-risk HPV, and an equivalent sensitivity for detection of histologically confirmed CIN2 or higher (92% using the device, compared with 95% using the clinician-collected specimen) (Brink et al., 2006).<br><br>Initial studies with the Pantarhei Screener have also shown that women find the device easy-to-use. In two pilot studies in the Netherlands, in which 300 women who had missed their standard pap screening, received the Screener at home, almost all participating women reported that using the Screener was easy. In response to these findings, an ongoing study approved by the Dutch National Health Authority includes mailing a user-friendly, self-sampling kit with the Pantarhei Screener for HPV testing to 29,000 women in the Netherlands who did not respond to the invitation twice in 2005. The self-collected Screener specimens are being tested for high-risk HPV using the Digene Hybrid Capture II test, with positive women followed for clinical evaluation. Preliminary results show that approximately 7,400 women (30%) have used the

Columbia University IRB
Approved for use until: 04/09/2010

	Screener and mailed in their specimens, with approximately 10% positive for high-risk HPV, and four cancers already identified.
Device version number:	not applicable
Sponsor Protocol version number:	
Significant risk:	No
Justification of non significant risk:	The device is atraumatic (has no sharp edges) and is similar to a plastic tampon applicator. (Please see the photograph of the device attached to this submission). It contains buffered saline which is known to be a body-friendly fluid. This is the same fluid commonly used in eyewash. The device has been used extensively in Europe for HPV testing. The Dutch National Health Authority sent the device home to approximately 29,000 women who had not responded to their cervical cancer screening program for the women to self-collect specimens at home and mail them to a laboratory. Over 7,000 women used the device and returned their specimen; with no reports of Adverse Events from using the device.<br><br>Furthemore, women in the US are currently allowed to self-collect vaginal swabs (long q-tip like swabs), as the FDA has cleared self-use of vaginal swabs for detection of sexually transmitted infections such as chlamydia. We believe use of the Pantarhei Screener is analogous to self-use of a vaginal swab and thus is of non-significant risk to the user. If the device is found to provide valid specimens for cytology and/or HPV diagnostics, the sponsor will use study results to seek FDA clearance for the device.
Drug dosage:	Not applicable
Drug dosage unit:	Not applicable
Drug administration route:	
IDE/IND Holder Type:	
IDE/IND Holder:	
IDE/IND number:	not applicable
Does the IDE/IND Number begin with the letter G?	No
Is this device a Carotid Stent?	No
Explanation of IND amendment:	
Phase of study:	Phase II
Manufacturer:	Pantarhei Devices BV<br>Rene Hol<br>PO Box 464<br>3700 AL Zeist, 3700 AL
Contact phone:	31-30-6-985-211
Contact Email:	rh@pantarhei-devices.com

Human Specimen

<u>Type</u>	<u>Origin</u>	<u>Description</u>
Fluid	Biopsy	Women will collect their own vaginal lavages using the

Columbia University IRB
Approved for use until: 04/09/2010

Pantarhei Screener, a self-sampling device, containing 5 ml of buffered saline.

Source of specimen:	The woman will collect the specimen herself.
Method of obtaining:	The specimens will be collected specifically for this study.
Length of retention:	2 years
How donor will be identified:	The specimens will be identified by the woman's study ID number and her initials. The initials will be used to verify that the correct ID number was used, without jeopardizing her confidentiality.
Specimens will be reused:	Yes

Attached Hazardous Material Reports

Type	Appendix Number
Human blood, all human cell lines or other potentially infectious materials (OPIM)	APC-AAAA2820

Attached Documents

Document Identifier	File Name	Active	Archived	Date Attached
Medical Devices Complaint Form v3.0	PD cyt feas_Med Complaint CRF Eng_v3.0_08Oct28.doc	Y	N	10/28/2008
Eligibility Checklist v3.0	PD cyt feas_Elig Checklist CRF Dual_v3.0_08Oct28.doc	Y	N	10/28/2008
Telephone script English v3.0	PD cyt feas_Phone Script Eng_v3.0_08Oct28.doc	Y	N	10/28/2008
End of Study Results Form v3.0	PD cyt feas_End of Study CRF Eng_v3.0_08Oct28.doc	Y	N	10/28/2008
Recruitment Letter IDC	PD cyt feas_Recruitment LetterDual_KB.pdf	Y	N	10/28/2008
Recruitment Flyer IDC	PD cyt feas_IDC recruitment flier_v3.0_08Oct28.doc	Y	N	10/28/2008
Telephone script Spanish v3.0	PD cyt feas_Phone Script Span_v3.0_08Oct28.doc	Y	N	10/28/2008
Approval Letter Student Health Services	PD cyt feas_IRB Letter_SHS Wheat.pdf	Y	N	04/17/2009
Approval Letter FPPS Private Office HIP	PD cyt feas_IRB Letter_HIP Westhoff.pdf	Y	N	04/17/2009
Recruitment Letter SHS	PD cyt feas_Recruitment Letter Eng_Wheat.pdf	Y	N	04/17/2009
Demographic and Acceptability Form v3.0	PD cyt feas_Demog Accept CRF Dual_v3.0_08Oct28.doc	Y	N	10/28/2008
User instructions English v3.0	PD cyt feas_User Instruction Screener Eng_v3.0_08Oct28.pub	Y	N	10/28/2008
Photo of Screener v3.0	PD cyt feas_Photo of the Pantarhei Screener_v3.0_08Oct28.doc	Y	N	10/28/2008
User instructions Spanish v3.0	PD cyt feas_User Instruction Screener Span_v3.0_08Oct28.pub	Y	N	10/28/2008
Medical Devices Near Incident Form v3.0	PD cyt feas_Med Device (Near) Incident CRF Eng_v3.0_08Oct28.doc	Y	N	10/28/2008
Full protocol v3.0	PD cyt feas_Full Protocol_v3.0_08Oct28.doc	Y	N	10/28/2008
Approval letter Associates for Internal Medicine	PD cyt feas_IRB Letter_AIM Lantigua.pdf	Y	N	04/09/2008
Staff Observation Form v3.0	PD cyt feas_Staff Obs CRF Eng_v3.0_08Oct28.doc	Y	N	10/28/2008

Columbia University IRB
Approved for use until: 04/09/2010

Index Cytology Form v3.0	PD cyt feas_Index Cytology CRF Eng_v3.0_08Oct24.doc	Y	N	10/28/2008
Colposcopy Results Form v3.0	PD cyt feas_Colpo CRF Eng_v3.0_08Oct28.doc	Y	N	10/28/2008
Recruitment Letter AIM	PD cyt feas_Recruitment Letter Dual_RL.pdf	Y	N	10/28/2008
Approval letter Family Planning Clinic	PD cyt feas_IRB Letter_FPC Westhoff.pdf	Y	N	04/09/2008
Pre Screener Acceptability Form v3.0	PD cyt feas_Pre Accept CRF Dual_v3.0_08Oct28.doc	Y	N	10/28/2008
Recruitment Letter FPC	PD cyt feas_Recruitment Letter Dual_CW.pdf	Y	N	10/28/2008
Screener Cytology Form v3.0	PD cyt feas_Screener Cytology CRF Eng_v3.0_08Oct28.doc	Y	N	10/28/2008
Approval Letter Infectious Disease Clinic	PD cyt feas_IRB Letter_IDC Brudney.pdf	Y	N	04/09/2008
Spanish translation memo v4.0	PD cyt feas_Spanish Memo_v4.0_09May27.pdf	Y	N	05/27/2009
Screening Form v4.0	PD cyt feas_Screen CRF Dual_v4.0_09May27.doc	Y	N	05/27/2009
Spanish Informed Consent form v4.0	PD cyt feas_Informed Consent Span_v4.0_09Jun26.doc	Y	N	06/26/2009

Attached Consent Forms

Title	Consent Number	Active	Initiator
Pantarhei Screener cytology feasibility and acceptability	CF-AAAE6391	Active	Jones

Attached HIPAA Forms

View Form	Title	Initiator	Date Created
HIP-AAAA9457	Form A: HIPAA Clinical Research Authorization for Sponsored Research	Jones, Heidi	03/12/2008
HIP-AAAB0047	Formulario A: Autorización de la Ley HIPAA para la investigación clínica patrocinada	Jones, Heidi	05/13/2008

Approval Personnel

Electronic Signature: Karen Brudney (527) - Co-Investigator	Date: 03/12/2009
Electronic Signature: Michelle DiVito (538) - Department Administrator	Date: 03/13/2009
Electronic Signature: Heidi Jones (538) - Co-Investigator	Date: 03/12/2009
Electronic Signature: Rafael Lantigua (527) - Co-Investigator	Date: 03/12/2009
Electronic Signature: Mahesh Mansukhani (548) - Co-Investigator	Date: 03/15/2009
Electronic Signature: Irene Raju (538) - Study Coordinator	Date: 03/12/2009
Electronic Signature: Guo-Xia Tong (548) - Co-Investigator	Date: 03/13/2009
Electronic Signature: Carolyn Westhoff (538) - Principal Investigator	Date: 06/26/2009
Electronic Signature: Tasha Smith (512) - Cancer Center	Date: 06/08/2009
Electronic Signature: Mary Wheat (527) - Co-Investigator	Date: 06/01/2009
Electronic Signature: Teresa Wood (548) - Co-Investigator	Date: 03/16/2009

Columbia University IRB
Approved for use until: 04/09/2010

Columbia University Human Subjects Study Description Data Sheet

Protocol: IRB-AAAD1382(Y2M01) Modification | **Protocol Status: Approved** | **Effective Date: 07/09/2009**
Expiration Date: 04/09/2010

Originating Department: **OBSTETRICS AND GYNECOLOGY (538)**
Submitting To: **Medical Center**
Title: **The feasibility and acceptability of using the Pantarhei Screener for cervical cytology testing among low income women in New York City**
Sponsor Protocol Version#:
Abbreviated title: **Feasibility and acceptability of the Screener for cytology**
IRB of record: **Columbia University Medical Center**
IRB number used by the IRB of record:

Affiliated Institutions: -Standard Columbia Submission
Protocol Begin Date: 05/01/2008
Protocol End Date: 09/30/2009

Principal Investigator: Carolyn Westhoff (538)

Study Description

1. Study Purpose and Rationale

Cervical cancer is among the top ten cancers identified in Black and Hispanic women in New York State (CDC, 2002). In New York City, women from low income, predominantly African American and Hispanic neighborhoods have the highest rates of cervical cancer (NYC DOH, 2003).

Cervical cancer screening relies on providers obtaining cervical cells during a speculum exam. Cervical cell collection (often referred to as a 'Pap smear') requires significant infrastructure and time; is an invasive, often negatively perceived experience for women; and is difficult to integrate into primary health care settings, resulting in referrals with high loss to follow-up rates.

Women who have completed childbearing often do not get screened because they do not like the pelvic exam (Matseoane & Westhoff, 2000). A preliminary audit of two primary health clinics in the Columbia University Medical Center show women often wait 2-3 months for cervical cancer screening because of insufficient availability of providers. A recent project aimed at disseminating cervical cancer screening guidelines to primary care providers in northern Manhattan and the South Bronx found that more than half of the providers in the study did not offer cervical cancer screening services themselves; yet, there are few local obstetricians and gynecologists for referrals, reducing the likelihood of women receiving Pap smears. Similarly, most women in in-patient settings within the Columbia Presbyterian Hospital do not agree to a Pap test if they are due to be screened, resulting in many missed opportunities for screening.

This study will assess the feasibility (in terms of preliminary validity data) and acceptability of using a new self-collection device, the Pantarhei Screener, for cervical cytology readings. Primary and tertiary care settings are missing many opportunities to screen women for cervical cancer. Should the Screener prove acceptable and valid for cytology, further studies will validate the device for human papillomavirus (HPV) and will test whether the device can be integrated into services to decrease missed opportunities for screening services in our community, with applications to a wide array of healthcare settings. Use of the Screener does not require a speculum exam or clinician participation.

2. Study Design and Statistical Procedures

A single center, cross-sectional study comparing cytology results from self-administered Pantarhei Screener specimens with cytology results from clinician-obtained endocervical spatula with brush specimens in 210 women with recent

Columbia University IRB
Approved for
use until: 04/09/2010

(1-3 months prior to enrollment) Pap smears from the five participating clinics (Family Planning Clinic, Infectious Diseases Clinic, Associates for Internal Medicine, Family Planning & Preventive Services private offices, Student Health Services). Women will receive the Screener device and user instructions at their study visit for self-collection of a vaginal lavage. They will answer a brief acceptability questionnaire prior to using the device (see pre-sampler acceptability form). After using the Screener, women will answer questions on acceptability, including any discomfort from the study procedure, and understanding of user instructions, as well as key socio-demographic information. Screener specimens from women whose original cytology smear was tested for HPV, will also be tested for HPV. Additionally, the results for women who receive colposcopy will be recorded as secondary endpoints.

For the primary objective of the study, we will calculate the kappa statistic (with 95% confidence intervals) and percent agreement (with 95% confidence intervals) between the original Pap results and the results using the Pantarhei Screener, using dichotomous classification of Pap smears as normal or abnormal. To determine sensitivity, and specificity between the two collection methods, the previously collected cytology results using the clinician-collected endocervical specimen with ThinPrep will be defined as true cases. To compare sensitivity and specificity to detect HSIL lesions between the two collection methods, histologically confirmed lesions from colposcopy and biopsy will be defined as true cases.

As the study's primary aim is to determine feasibility, the sample size has been calculated to provide sufficient data to determine whether a larger study is needed to validate use of the Screener for cytology and to seek US FDA clearance. To detect a sensitivity/specificity of 80% with a lower 95% confidence limit of 65%, we would need 98 cases (Flahuault et al. 2005). We will therefore enroll 200 women100 with a recent (last 1-3 months) normal Pap and 100 with a recent abnormal Pap (as defined by current Presbyterian Hospital Guidelines). Additionally 10 women will be enrolled to pilot the questionnaires as well as the laboratory processes, for a total of 210 women to participate in the study.

3. Study Procedures

We will recruit women as outlined in the recruitment section below. Interested women will come to the clinical research facility at the Division of Family Planning & Preventive Services in the Presbyterian Hospital's Department of Obstetrics & Gynecology, for their single study visit, pending any abnormalities.

Upon arrival at the site, women will complete an eligibility checklist. If they are eligible, they will read the informed consent form. Women interested in participating after reading the informed consent form will have a private discussion with a research assistant to answer any questions the woman may have about the study. After signing the informed consent form, the woman will be given a sealed Pantarhei Screener with user instructions. She will then answer a brief acceptability questionnaire prior to using the Sampler. Next, she will go to a designated examination room to collect her specimen. When she has completed the specimen collection, she will meet with the research assistant to be interviewed on socio-demographics and acceptability questions. Additionally, staff will be available to provide counseling on abnormal Pap results should the woman have questions about her original Pap results. Finally, the research assistant will assist women with abnormal results in making appointments with the colposcopy clinic, further encourage them to maintain existing appointments, or arrange for them to return to the research site to receive colposcopy. The visit should take no longer than 60 minutes.

Clinical management of women will be based on their previously obtained cytology results, unless the Screener is found to be abnormal and the Pap is normal; these women will be invited for colposcopy by clinic staff to rule out the possibility of a false negative Pap smear, and ensure patient safety. In this scenario, research staff will send the new abnormal results to the Medical Director of the clinic where the woman obtained her original cytology screening for follow-up per clinic protocol. The costs of Pap smear and/or colposcopy visits due to an abnormal result using the investigational device will be paid for by the research budget; women will not be billed for these procedures.

4. Pantarhei Device

The Pantarhei Screener is a sterile, plastic, syringe-like device containing 5 mls of buffered saline that allows a woman

Columbia University IRB
Approved for
use until: 04/09/2010

to collect her own vaginal lavage (see attached photo of device). The device was designed by gynecologists to fit a woman's body. The atraumatic shape precludes the potential for injuries from using the device, making it a device with non-significant risk. The Screener currently has a CE label for Class 1 sterilized products allowing it to be used on a commercial basis in Europe and many countries in Asia.

Data to date suggest that the device provides a high quality specimen for the detection of high-risk HPV. In a study among 96 women in the Netherlands, the Screener had 87% agreement with a kappa of 0.71 compared with an endocervical brush obtained by a clinician in combination with the GP5+/6+ polymerase chain reaction (PCR) test to detect high-risk HPV, and an equivalent sensitivity for detection of histologically confirmed CIN2 or higher (92% using the device, compared with 95% using the clinician-collected specimen) (Brink et al., 2006).

Initial studies with the Pantarhei Screener have also shown that women find the device easy-to-use. In two pilot studies in the Netherlands, in which 300 women who had missed their standard Pap screening, received the Screener at home, almost all participating women reported that using the Screener was easy. In response to these findings, an ongoing study approved by the Dutch National Health Authority includes mailing a user-friendly, self-sampling kit with the Pantarhei Screener for HPV testing to 29,000 women in the Netherlands who did not respond to the invitation twice in 2005. The self-collected Screener specimens are being tested for high-risk HPV using the Digene Hybrid Capture II test, with positive women followed for clinical evaluation. Preliminary results show that approximately 7,400 women (30%) have used the Screener and mailed in their specimens, with approximately 10% positive for high-risk HPV, and four cancers already identified. No data exists, however, on the use of the Screener for cytology using ThinPrep medium.

5. Study Questionnaires of Case Record Forms (CRFs)

The following CRFs will be used for the study (also attached):

A. Screening Form - This CRF will be used to ensure participants meet the eligibility criteria before making an appointment.

B. Eligibility Checklist - This CRF will be used to ensure participants meet the eliglibility requirements at the study visit.

C. Pre-Screener Acceptability Form - This CRF will be used to elicit how the woman imagines using the Screener and following the instructions will be, prior to use.

D. Demographic and Acceptability Form - This CRF will be used to elicit participant experiences with use of the device and user instructions after having used the device, as well as key socio-demographic characteristics.

E. Staff Observation Form - This CRF will be completed by study staff to note any participant questions about use of the device and/or user instructions during the study visit.

F. Medical Devices Complaint Form - This CRF will be used to report any untoward medical complaint in a participant who used the device and which does not necessarily have to be related to the investigational device. Whether a medical complaint is entered on the Medical Devices Complaint Form depends on whether it is clinically relevant according to the investigator.

G. Medical Devices (Near) Incident Form - This CRF will be used to report any malfunction or deterioration in the characteristics and/or performance of a device, as well as any inadequacy in the labelling or the instructions for use which might lead to or might have led to a Serious Adverse Event.

H. Screener Cytology Results Form - This CRF will be completed by laboratory staff during processing of specimens.

I. Index Cytology Form - This CRF will be completed by study staff by accessing WebCis to record the results from

Columbia University IRB
Approved for use until: 04/09/2010

the original cytology test.

J. Colposcopy Results Form - This CRF will be completed by study staff by accessing WebCis within 6 months of the original cytology for women with abnormal cytology readings.

6. Recruitment

We will recruit and enroll women for three to six months. Based on current patient statistics, we estimate 40 women will participate per month.

Doctors from the participating clinics will provide us with a list of patients with recent Pap smears who can be invited to participate. These women will have received their Pap results in the mail, per standard clinic procedures. Additionally, the Medical Director of each clinic will mail a separate letter informing them that they may be invited to participate in this study and that participation is voluntary (see attached letter). After they have received this letter, we will phone these women and invite them to participate in the study, as well as screen for initial eligibility criteria (see attached screening form). Interested women will make appointments to come to one of the research sites. At the site, they will be asked eligibility criteria (see attached eligibility checklist). If they are still eligible, they will read the informed consent form. Women interested in participating after reading the informed consent form will have a private discussion with a research assistant to answer any questions the woman may have about the study.

7. Study Participants

Eligibility criteria for participation are outlined below:

Inclusion criteria
- Valid Pap smear in last 1-3 months obtained at participating clinic
- 18 years or older
- Self-report being able to read in English and/or Spanish
- Willing to sign informed consent

Exclusion criteria
- Used vaginal product (douche, spermicide, antifungal) in last 48 hours
- Last menses started less than or equal to 4 days prior to enrollment visit
- No uterus / history of hysterectomy
- Self-report currently pregnant
- Self-report currently breastfeeding
- No colposcopy appointment between original pap smear and study visit

Women with menses who are interested in participating in the study will return after menses has completed to undergo study screening and informed consent. Women will be scheduled after at least one month has passed from their original Pap smear exam and prior to any subsequent colposcopy exams to ensure adequate replacement of cells for collection from the cervix. Ideally, women will be scheduled between 1 and 2 months after their initial specimen collection to allow for sufficient replacement of cells while avoiding real changes in cervical status; however the upper limit will be 3 months to facilitate study logistics. The time interval between collections will be recorded for analysis.

8. Confidentiality of Study Data

All interviews will be conducted in private. All case record forms (CRFs), other study documents, and specimens will be identified by ID numbers and initials only, to maintain participant confidentiality. Both an ID number and initials will be used to verify that the participant has been correctly identified for this clinical trial.

Study staff will keep individual contact sheets, containing the name, medical record number, study ID number, initials, and date-of-birth of each woman, as well as her contact information. This contact sheet, a master subject log and the

Columbia University IRB
Approved for
use until: 04/09/2010

signed Informed Consent Form will be the only means of linking ID numbers with names and contact information. They will therefore be kept locked in a separate file from the CRFs.

Study data that are transmitted electronically will not contain any personal identifying information. While regulatory agencies and the sponsor may request access to study records, the identity of subjects will always remain confidential to the fullest extent possible by law. Information will not be released without the permission of the participant.

9. Potential Risks

There are minimal risks to the participants. However, there may be slight discomfort from using the device, such as a delayed release of a slight amount of retained fluid. The atraumatic shape of the device and clear user instructions should minimize potential risks. To date, no adverse events have been reported from using the device extensively (in over 7000 women) in Europe.

10. Potential Benefits

The benefit for women with abnormal Pap results will be further counseling on the meaning of their result, and assistance in arranging further assessment through colposcopy visits. A review of current records of women with abnormal Pap results in the study clinics suggests that high rates of these women do not maintain colposcopy appointments for further assessment. Additional counseling and assistance in setting up the appointments should improve the rates of follow-up. There is no direct benefit to women with normal Pap results. Should this method prove to be feasible, it could improve access to cervical cancer screening for women in these clinics in the future.

11. Alternatives

Participation will be voluntary; women can choose not to participate with no impact on their clinical care or services to which they are entitled.

12. References

Brink AATP, Meijer CJLM, Wiegerinck MAHM, et al. High concordance of results of testing for human papillomavirus in cervicovaginal samples collected by two methods, with comparison of a novel self-sampling device to a conventional endocervical brush. J Clin Microb 2006;44:2518-33.

Centers for Disease Control & Prevention (CDC). 2002 Top 10 cancers by geographic area. http://apps.nccd.cdc.gov/uscs/Table.aspx?Group=3f&Year=2002&Display=n. Accessed on 8/25/2006.

Flauhault A, Cadilhac M, Thomas G. Sample size calculation should be performed for design accuracy in diagnostic test studies. J Clinic Epi 2005l58:859-62.

Matseoane D, Westhoff C. Papanicolaou screening after postpartum tubal ligation. Presentation at the Annual Association of Reproductive Health Professionals Conference, La Jolla, CA, 2003. Abstract published in Contraception 2003; 68(2):145.

New York City Department of Health. Cancer incidence and mortality by NYC neighborhood, 1999-2003, New York State. http://www.health.state.ny.us/statistics/cancer/registry/table5. Accessed on 8/25/2006.

Columbia University IRB
Approved for use until: 04/09/2010
