# Supplementary material for: Validity and Reliability of Using a Self-Lavaging Device for Cytology and HPV Testing for Cervical Cancer Screening: Findings from a Pilot Study
Source: PLoS One. 2013 Dec 20;8(12):e82115. doi: 10.1371/journal.pone.0082115 (PMC3869665; doi:10.1371/journal.pone.0082115)
Supplement: Checklist S1 — STARD Checklist for reporting of studies of diagnostic accuracy. (PDF) [file pone.0082115.s003.pdf]

STARD checklist for reporting of studies of diagnostic accuracy
(version January 2003)

Validity of using a self-lavaging device for cytology and HPV testing for cervical cancer screening: Findings from a pilot study / Jones, HE et al.

Section and Topic	Item #		Completed
TITLE/ABSTRACT/ KEYWORDS	1	Identify the article as a study of diagnostic accuracy (recommend MeSH heading 'sensitivity and specificity').	Yes used term 'validity'
INTRODUCTION	2	State the research questions or study aims, such as estimating diagnostic accuracy or comparing accuracy between tests or across participant groups.	Done
METHODS			
Participants	3	The study population: The inclusion and exclusion criteria, setting and locations where data were collected.	Done
	4	Participant recruitment: Was recruitment based on presenting symptoms, results from previous tests, or the fact that the participants had received the index tests or the reference standard?	Done
	5	Participant sampling: Was the study population a consecutive series of participants defined by the selection criteria in item 3 and 4? If not, specify how participants were further selected.	Done
	6	Data collection: Was data collection planned before the index test and reference standard were performed (prospective study) or after (retrospective study)?	Done
Test methods	7	The reference standard and its rationale.	Done
	8	Technical specifications of material and methods involved including how and when measurements were taken, and/or cite references for index tests and reference standard.	Done
	9	Definition of and rationale for the units, cut-offs and/or categories of the results of the index tests and the reference standard.	Done
	10	The number, training and expertise of the persons executing and reading the index tests and the reference standard.	Did not include the number for each test but described how cytology was performed
	11	Whether or not the readers of the index tests and reference standard were blind (masked) to the results of the other test and describe any other clinical information available to the readers.	Done
Statistical methods	12	Methods for calculating or comparing measures of diagnostic accuracy, and the statistical methods used to quantify uncertainty (e.g. 95% confidence intervals).	Done
	13	Methods for calculating test reproducibility, if done.	Done
RESULTS			
Participants	14	When study was performed, including beginning and end dates of recruitment.	Included in methods
	15	Clinical and demographic characteristics of the study population (at least information on age, gender, spectrum of presenting symptoms).	Done (in text and Table 1)
	16	The number of participants satisfying the criteria for inclusion who did or did not undergo the index tests and/or the reference standard; describe why participants failed to undergo either test (a flow diagram is strongly recommended).	Done (in text and Figure S1)

Test results	17	Time-interval between the index tests and the reference standard, and any treatment administered in between.	Done
	18	Distribution of severity of disease (define criteria) in those with the target condition; other diagnoses in participants without the target condition.	Done
	19	A cross tabulation of the results of the index tests (including indeterminate and missing results) by the results of the reference standard; for continuous results, the distribution of the test results by the results of the reference standard.	Done (Table 2)
	20	Any adverse events from performing the index tests or the reference standard.	Not applicable
Estimates	21	Estimates of diagnostic accuracy and measures of statistical uncertainty (e.g. 95% confidence intervals).	Done (in text and Table 3)
	22	How indeterminate results, missing data and outliers of the index tests were handled.	Done (in text and Figure S1)
	23	Estimates of variability of diagnostic accuracy between subgroups of participants, readers or centers, if done.	Not applicable
	24	Estimates of test reproducibility, if done.	Not applicable
DISCUSSION	25	Discuss the clinical applicability of the study findings.	Done
